# Efficacy of e‐learning using video content in improving trainees’ biliary cannulation skills and understanding (with video)

**DOI:** 10.1002/deo2.70068

**Published:** 2025-01-28

**Authors:** Junichi Kaneko, Yosuke Kobayashi, Masaki Takinami, Masaharu Kimata, Masafumi Nishino, Yurimi Takahashi, Yashiro Yoshizawa, Go Murohisa, Yoshisuke Hosoda, Takanori Yamada

**Affiliations:** ^1^ Department of Gastroenterology Iwata City Hospital Shizuoka Japan; ^2^ Department of Gastroenterology Seirei Hamamatsu General Hospital Shizuoka Japan; ^3^ Department of Hepatology Iwata City Hospital Shizuoka Japan

**Keywords:** biliary cannulation, e‐learning, education, endoscopic retrograde cholangiopancreatography, endoscopy training

## Abstract

**Objectives:**

E‐learning with video content was created to improve trainees’ biliary cannulation techniques; this study aimed to evaluate its educational effect prospectively.

**Methods:**

E‐learning program was conducted using videos demonstrating biliary cannulation for 24 papillae, targeting trainees with 2–6 years of experience in endoscopic retrograde cholangiopancreatography. Ten consecutive cases of biliary cannulation for native papillae performed by trainees were prospectively assessed before and after the e‐learning, respectively. The primary outcome was the difficult biliary cannulation rate; the secondary outcomes included a comprehension score assigned by the trainer for each biliary cannulation (maximum of 6 points), trainee failure rate, and adverse events incidence.

**Results:**

Eleven trainees participated in the e‐learning program. The overall and per‐trainee analyses showed no significant differences in the difficult biliary cannulation rate, trainee failure rate, and adverse event incidence before and after e‐learning. However, the overall analysis showed a significant increase in comprehension scores after e‐learning (median 4 vs. 5, *p *< 0.01) and the per‐trainee analysis revealed that the rate of comprehension score ≥5 increased significantly after e‐learning (*p *= 0.02). Comprehension score <5 (odds ratio: 4.31, *p *< 0.01) and endoscopic retrograde cholangiopancreatography experience <3 years (odds ratio: 2.15, *p = *0.01) were independent risk factors for difficult biliary cannulation. Additionally, the difficult biliary cannulation incidence showed a negative correlation with the comprehension score (*p *< 0.01).

**Conclusions:**

E‐learning using video content did not result in a reduction in the difficult biliary cannulation rate. However, it significantly enhanced procedural understanding, indicating its potential to support future acquisition of biliary cannulation skills.

## INTRODUCTION

Biliary cannulation through the major duodenal papilla (MDP) is an essential technique in endoscopic retrograde cholangiopancreatography (ERCP). However, biliary cannulation remains challenging for many gastrointestinal endoscopists, largely due to its failure rate of 5%–15%.^1^, ^2^ ERCP trainees must gain substantial experience in biliary cannulation to improve their skills. However, studies show that even after 200–400 procedures, the success rate is only 80%–82%.^3^, ^4^ Experience alone may not suffice for satisfactory skill improvement.^5^ Mechanical simulators can aid in technique refinement,^6^, ^7^, ^8^, ^9^ but access is limited. Therefore, tools that enable trainees to learn biliary cannulation anywhere and in any situation are needed.

The urgency for introducing these learning tools was exacerbated by the coronavirus disease 2019 (COVID‐19) pandemic. COVID‐19, caused by severe acute respiratory syndrome coronavirus 2, imposed widespread restrictions on endoscopic practice to prevent COVID‐19 outbreaks.^10^, ^11^ Therefore, during the pandemic, trainees had limited opportunities to learn biliary cannulation in the endoscopy suite, and tools were needed to enable learning biliary cannulation without human contact.^10^, ^11^, ^12^

We produced video content on biliary cannulation and provided it to trainees via the Internet as an e‐learning program. This e‐learning program allowed trainees to learn biliary cannulation anywhere and in any situation without human contact. The present study aimed to evaluate the effectiveness of e‐learning with video content in a multicenter setting.

## METHODS

### Study design

This prospective, multicenter, observational study was conducted at two general hospitals in Shizuoka, Japan (namely, Iwata City Hospital and Seirei Hamamatsu General Hospital) from June 2021 to October 2022. This study was approved by the institutional review board of each participating center (approval no of the Institutional Review Board: 2022–038 [Iwata City Hospital] and 3544 [Seirei Hamamatsu General Hospital]). Written informed consent was obtained from all participating trainees. Consent for the use of patient information was obtained through an opt‐out system. This study was registered in the University Hospital Medical Information Network Trials Registry (UMIN000044223).

### E‐learning with video content

**FIGURE 1:**
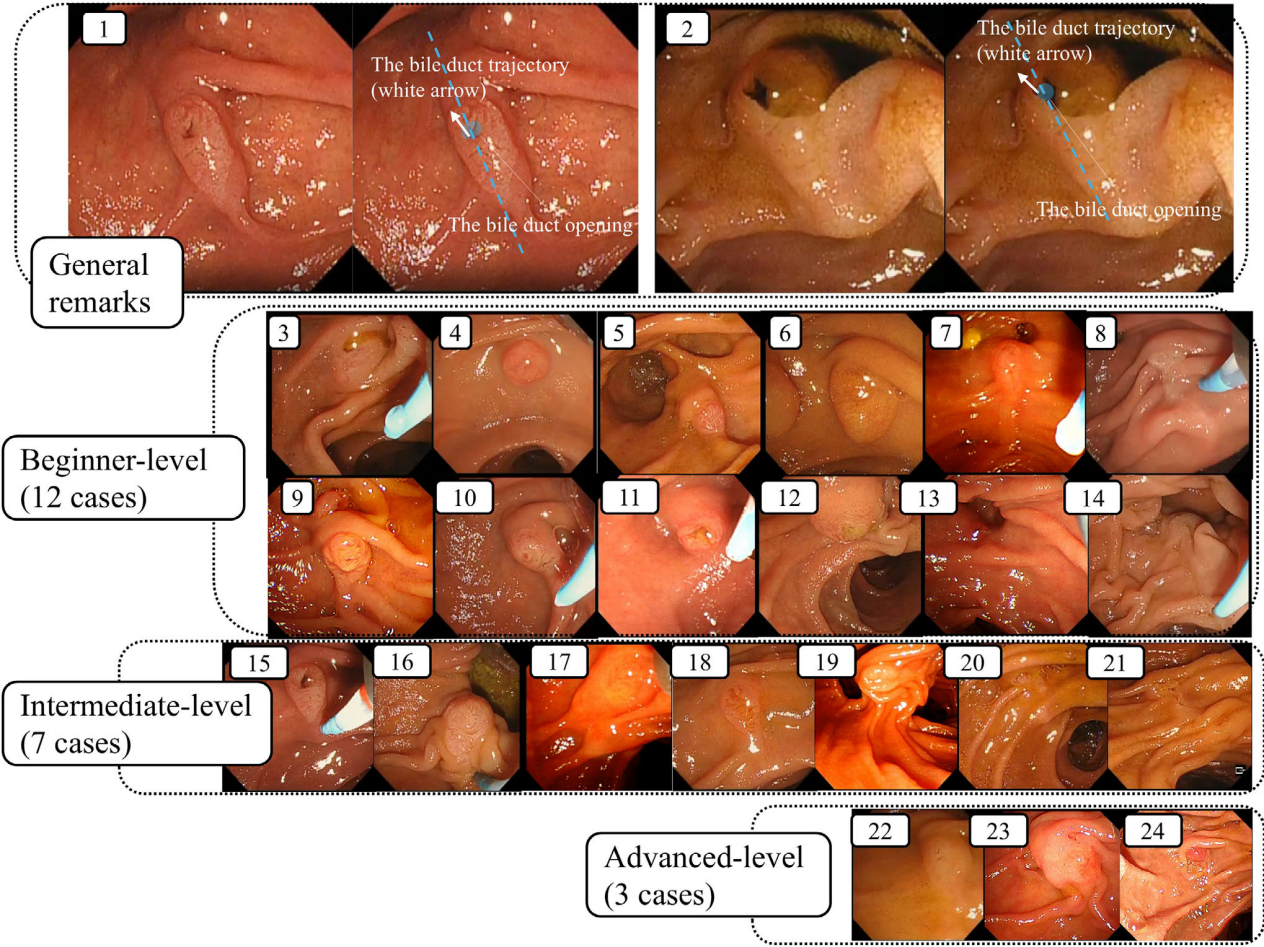
All major duodenal papillae presented in the e‐learning video content: No. indicates the video number.

The video content was produced by JK and YK, who are certified specialists in gastroenterological endoscopy by the Japan Gastroenterological Endoscopy Society and have experience with > 2000 ERCP cases. First, 24 videos were selected for educational purposes from the cases involving biliary cannulation performed on patients with native papillae at our two hospitals. Subsequently, only the endoscopic and fluoroscopic scenes related to biliary cannulation were extracted from the videos, and a 3–5‐min video was created. All videos include an explanation of the frontal view of the MDP, the opening of the bile duct, and the course of the bile duct. Additionally, several videos provide technical tips for biliary cannulation specific to each MDP and an explanation of the difference between the pancreatic and bile duct trajectories. Finally, 24 e‐learning videos were completed, including two with general remarks, 12 at the beginner level, seven at the intermediate level, and three at the advanced level. The level of each video was determined at the discretion of the video producers, based on the complexity of biliary cannulation (Figure 1, Table 1, and Video S1).

**TABLE 1:** Structure and commentary for e‐learning videos.

Level	No.	Macroscopic appearance of the MDP	Comments on the knowledge and skills in each video
General remarks	1	Type 1	Basic explanation of the structure around the MDP and biliary cannulation
General remarks	2	Type 1	Basic explanation of the structure around the MDP and biliary cannulation
Beginner	3	Type 1	How to bring the endoscope closer to the MDP
Beginner	4	Type 1	How to bring the endoscope closer to the MDP
Beginner	5	Type 3	Sense of the distance between the endoscope and MDP
Beginner	6	Type 1	The difference in the course of the pancreatic duct and bile duct
Beginner	7	Type 1	Sense of the distance between the endoscope and MDP
Beginner	8	Type 1	The difference in the course of the pancreatic duct and bile duct
Beginner	9	Type 1	Sense of the distance between the endoscope and MDP
Beginner	10	Type 1	How to bring the endoscope closer to the MDP
Beginner	11	Type 3	How to bring the endoscope closer to the MDP
Beginner	12	Type 3	How to manipulate a cannula
Beginner	13	Type 2	Differences in the course of the pancreatic duct and bile duct in cases of duodenal diverticulum
Beginner	14	Type 1	Differences in the course of the pancreatic duct and bile duct in cases of duodenal diverticulum
Intermediate	15	Type 1	How to manipulate a cannula; exposing the MDP using a cannula
Intermediate	16	Type 1	How to manipulate a cannula; exposing the MDP using a cannula
Intermediate	17	Type 2	How to identify the structures surrounding the MDP
Intermediate	18	Type 1	The difference in the course of the pancreatic duct and bile duct
Intermediate	19	Type 3	How to identify the structures surrounding the MDP
Intermediate	20	Type 1	The difference in the course of the pancreatic duct and bile duct
Intermediate	21	Type 4	The difference in the course of the pancreatic duct and bile duct
Advanced	22	Type 1	How to manipulate a guidewire during difficult biliary cannulation
Advanced	23	Type 3	Double‐guidewire technique in difficult biliary cannulation
Advanced	24	Type 1	How to manipulate a guidewire during difficult biliary cannulation


*Note*: The macroscopic appearance of the MDP was classified into four categories according to the Scandinavian Society of Gastrointestinal Endoscopy Classification: regular (type 1), small (type 2), protruding or pendulous (type 3), and creased or ridged (type 4).

Abbreviation: MDP, major duodenal papilla.

### Study protocol

Endoscopy trainees who met all the following criteria were included: (1) 2–6 years of ERCP experience, and (2) working at the two participating hospitals and not planning to transfer to a hospital other than the two hospitals within 6 months of study participation.

**FIGURE 2:**
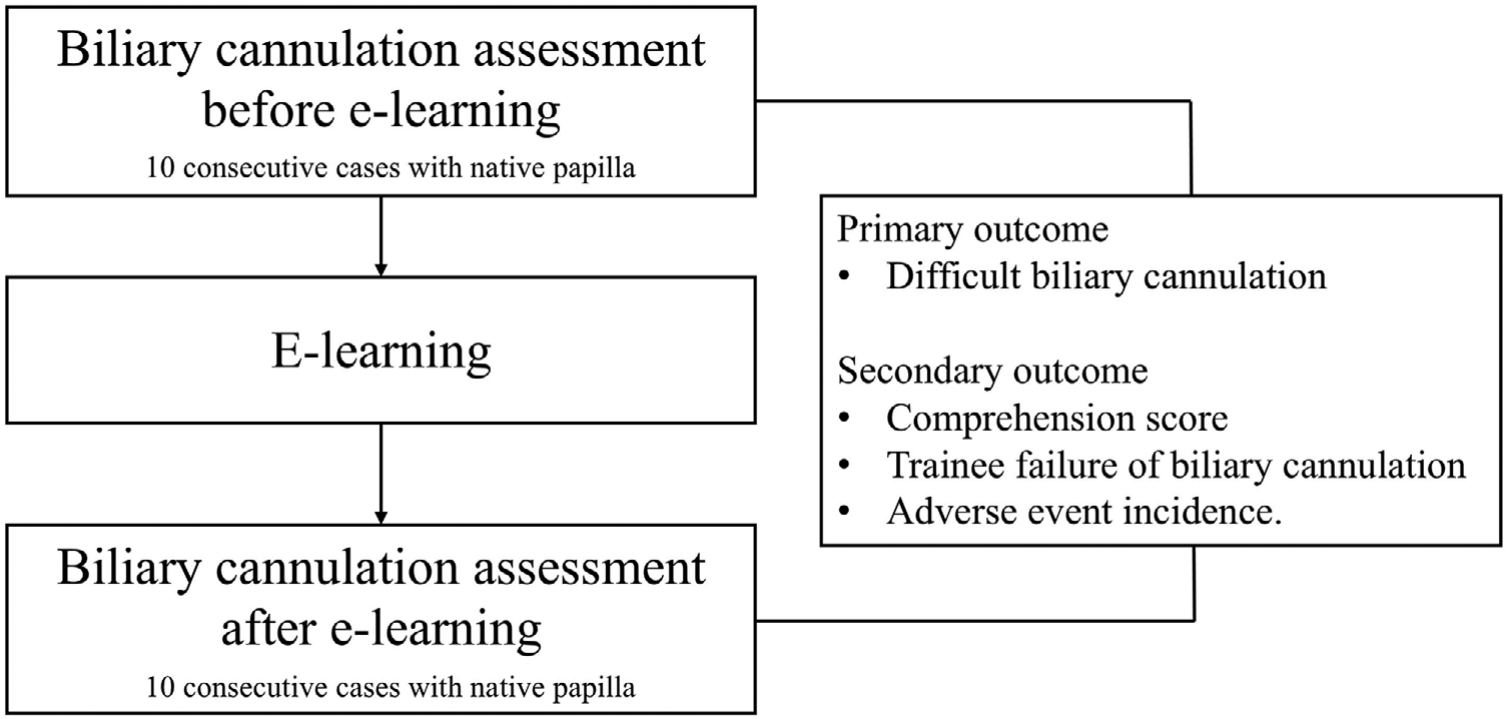
Schematic diagram of the research protocol.

First, each trainee performed biliary cannulation for 10 consecutive native papillae, and comprehension and technical outcomes were assessed. Subsequently, each trainee studied independently using e‐learning with video content. Finally, each trainee performed biliary cannulation for 10 consecutive native papillae, and comprehension and technical outcomes were assessed again (Figure 2). Trainees were required to watch all the videos at least once before taking the post‐e‐learning assessment; however, there was no restriction on the number of times they could view them. Patients with a history of upper gastrointestinal surgery other than Billroth I surgery were not included in pre‐ and post‐e‐learning assessments. The trainee performed biliary cannulation along with trainers who were endoscopists with experience in more than 1000 ERCP cases. Biliary cannulation was initiated using standard techniques, including contrast‐assisted and wire‐guided cannulation. The trainees were not advised by the trainers during the first 5 min of the biliary cannulation procedure. If more than 5 min elapsed or the patient's condition deteriorated, the trainers were allowed to advise and take over the procedure from the trainees.

### Outcome measure and definition

The primary outcome was a difficult biliary cannulation (DBC) rate to assess the biliary cannulation technique. The secondary outcomes were the comprehension score (CS), trainee failure of biliary cannulation (TF) rate, and adverse event (AE) incidence.

DBC was defined according to the following criteria: a trainee required ≥5 min of cannulation time, ≥5 attempts at biliary cannulation, or ≥2 pancreatic guidewire passages or contrast injections (5‐5‐2 criteria).^13^ Cannulation time was measured from the first contact with the papilla until successful biliary cannulation. Loss of contact and subsequent repositioning were counted as separate attempts. Pancreatic duct guidewire passages were counted, along with any partial or complete filling of the pancreatic duct by contrast regardless of intent. Successful biliary cannulation was defined as deep biliary cannulation with the guidewire or ERCP catheter positioned well inside the common bile duct. The CS was assigned by the trainer to the trainee via oral examination immediately before biliary cannulation (Table 2). The questions asked were about (1) the frontal view of the MDP, (2) the opening of the bile duct, and (3) the course of the bile duct. If the trainer and trainee were in complete agreement, the trainee received 2 points; if they were in partial agreement, the trainee received 1 point; and if they were not in agreement, the trainee received 0 points. Each of the three items was evaluated, and a maximum of 6 points was awarded for each case. TF was defined as the inability of a trainee to achieve successful biliary cannulation within 10 min using standard techniques. AEs were defined as any AE occurring within 2 days after ERCP. The severity of endoscopic AEs was graded based on the lexicon proposed by the American Society of Gastrointestinal Endoscopy.^14^ The macroscopic appearance of the MDP was classified into four categories according to the classification of the Scandinavian Association for Digestive Endoscopy: regular (type 1), small (type 2), protruding or pendulous (type 3), and creased or ridged (type 4).^15^ Haraldsson et al. showed that small type 2 papillae and protruding or pendulous type 3 papillae were more difficult to cannulate.^16^

**TABLE 2:** Comprehension score: Comprehension score was assigned by the trainer to the trainee via oral examination. Upon reaching the major duodenal papilla and immediately before initiating biliary cannulation, the trainer asked the following three questions to the trainee each time. The trainer assigned scores based on the degree of agreement between the trainer and the trainee.

	Complete agreement: points	Partial agreement: points	Disagreement: points
1. Please obtain a frontal view of the MDP using the endoscope.	2	1	0
2. Please indicate the opening of the bile duct.	2	1	0
3. Please explain the course of the bile duct.	2	1	0

For each biliary cannulation, the trainee was assigned a score ranging from 0 to 6 points.

Abbreviation: MDP, major duodenal papilla.

### Statistical analysis

Continuous variables are presented as medians and ranges or interquartile ranges (IQRs) and were compared using the Mann–Whitney U test. Categorical variables are presented as n (%) and were compared using Fisher's exact test. The chi‐square test was used to compare categorical variables among three or more groups.

First, primary and secondary outcomes were compared between pre‐ and post‐e‐learning groups using overall and per‐trainee analyses. Overall and per‐trainee analyses were done using Fisher's exact test and the Wilcoxon rank‐sum test, respectively. Second, factors affecting DBC were analyzed using a logistic regression model for the following seven variables: disease (benign vs. malignant), the macroscopic appearance of the MDP (type 2 or 3 vs. type 1 or 4), peripapillary duodenal diverticulum (yes vs. no), trainees’ ERCP experience (<3 years vs. ≥3 years), initial method of biliary cannulation (contrast‐assisted cannulation vs. wire‐guided cannulation), CS (<5 years vs. ≥5 years) and e‐learning (before vs. after). The factors with substantial impact (*p *< 0.2) in the univariate analysis were subsequently evaluated in a multivariate analysis. Third, correlations between the CS and DBC rates were analyzed using the Cochran–Armitage test for trends.

Statistical significance was set at *p*‐values <0.05 for all tests. All statistical analyses were performed using EZR (Saitama Medical Center, Jichi Medical University, Saitama, Japan), which is a graphical user interface for R (provided by The R Foundation for Statistical Computing, Vienna, Austria).

## RESULTS

### Characteristics of participating trainees

Eleven endoscopy trainees participated in this study. The median age of the trainees was 29 years (range: 29–35), comprising 11 males and one female trainee. Five trainees had 2–3 years of ERCP experience, four had 3–4 years, one had 4–5 years, and one had 5 years of experience.

### ERCP characteristics in the pre‐e‐learning and post‐e‐learning groups

A total of 220 patients with native papillae underwent biliary cannulation by trainees before and after e‐learning (110 patients before e‐learning, and 110 patients after). Patient characteristics are shown in Table 3. The macroscopic appearance of the MDP, types 1, 2, 3, and 4, were seen in 75, 19, 11, and five cases, respectively, in the pre‐e‐learning group and in 95, 3, 4, and eight cases, respectively, in the post‐e‐learning group (*p <* 0.01).

**TABLE 3:** Characteristics of patients in the pre‐ and post‐e‐learning groups.

	Pre‐e‐learning group (*n *= 110)	Post‐e‐learning group (*n *= 110)	*p*‐value
Age			
Median [IQR], years	78 [69–84]	75 [66–83]	0.19
Sex			
Female, *n*	50 (45%)	52 (47%)	0.89
Disease			
Malignant, *n*	31 (28%)	34 (31%)	0.77
Peripapillary duodenal diverticulum			
Yes, *n*	38 (35%)	39 (35%)	1.00
Macroscopic appearance of the MDP			
Type 1/2/3/4, *n*	75/19/11/5	95/3/4/8	<0.01
Initial BC method			
Wire‐guided, *n*	85 (77%)	96 (87%)	0.08
More than 10 min after switching to a trainer or switching to another BC method			
Yes, *n*	28 (25%)	18 (16%)	0.14
Procedure for the major duodenal papilla			
None/ES/EPBD/ES+EPLBD/Pre‐cut, *n*	33/74/0/2/1	35/71/1/1/2	0.87
Procedure after BC			
None/ PS or ENBD deployment/ MS deployment/Stone extraction, *n*	9/54/41/6	6/64/26/14	0.05
Use of rectal NSAIDs			
Yes, *n*	42 (38%)	46 (42%)	0.68
Prophylactic pancreatic stent placement			
Yes, *n*	10 (9%)	4 (4%)	0.17

Abbreviations: BC, biliary cannulation; ENBD, endoscopic nasobiliary drainage; EPBD, endoscopic papillary balloon dilation; EPLBD, endoscopic papillary large balloon dilation; ES, endoscopic sphincterotomy; IQR, interquartile range; MDP, major duodenal papilla; MS, metallic stent; NSAIDs, nonsteroidal anti‐inflammatory drugs; PS, plastic stent.

### Comparison of difficult biliary cannulation in the pre‐e‐learning and post‐e‐learning groups

A total of 220 cases were analyzed across the pre‐ and post‐e‐learning groups, of which 109 were DBC cases (58 and 51 in the pre‐ and post‐e‐learning groups, respectively). The overall analysis showed no significant difference in the DBC rate in the pre‐ and post‐e‐learning groups (53% vs. 46%, *p *= 0.42; Table 4). The most common reason for DBC in both groups was pancreatic guidewire passage or contrast injection ≥2 (48% and 45% in the pre‐ and post‐e‐learning group, respectively).

**TABLE 4:** Overall analysis outcomes of the pre‐ and post‐e‐learning groups.

	Pre‐e‐learning group (*n *= 110)	Post‐e‐learning group (*n *= 110)	*p*‐value
Comprehension score			
Median [IQR]	4 [3–5]	5 [4–6]	<0.01
Difficult BC			
Yes, *n*	58 (53%)	51 (46%)	0.42
Primary reason for difficult BC			
Time ≥5 min/Attempts ≥5/Pancreatic guidewire passages or contrast injection ≥2	12/18/28	12/16/23	0.97
Trainee failure of BC			
Yes, *n*,	41 (37%)	30 (27%)	0.15
AEs incidence			
Yes, *n* Severity, Mild/Moderate, *n*	9 (8%) 6/3	15 (14%) 14/1	0.28
PEP			
*n*	5	12	
Hemorrhage			
	2	2	
Others			
	2	1	

Abbreviations: AEs, adverse events; BC, biliary cannulation; IQR, interquartile range; PEP, post‐endoscopic retrograde cholangiopancreatography pancreatitis.

**FIGURE 3:**
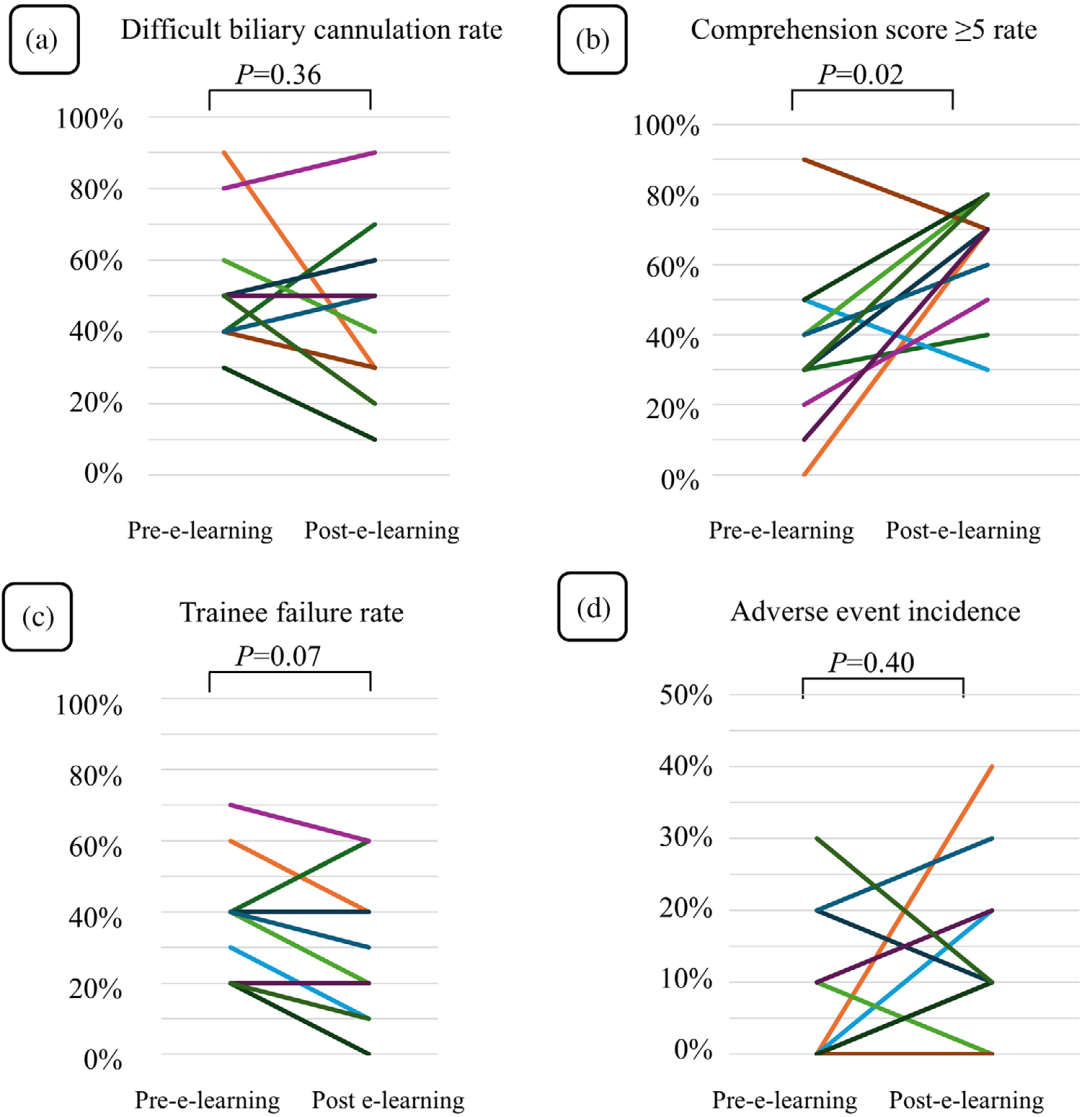
Graphs showing the analysis results for each trainee. (a) The rate of difficult biliary cannulation, (b) comprehension score, (c) the rate of trainee failure, and (d) adverse events incidence.

The per‐trainee analysis showed no significant reduction in the DBC rate after e‐learning (*p *= 0.36; Figure 3).

### Comparison of secondary outcomes in the pre‐e‐learning and post‐e‐learning groups

In the overall analysis, the median CS was significantly higher in the post‐e‐learning group than in the pre‐e‐learning group (4 [IQR 3–5] vs. 5 [IQR 4–6], *p *< 0.01); however, the TF rate (37% vs. 27%, *p *= 0.15) and AE incidence (8% vs. 14%, *p *= 0.28) did not significantly differ between the groups (Table 4).

In the per‐trainee analysis, the rate of CS ≥5 significantly increased after e‐learning (*p *= 0.02); however, the TF rate (*p *= 0.07) or AE incidence (*p *= 0.40) did not decrease significantly after e‐learning (Figure 3).

### Factors affecting difficult biliary cannulation

Univariate and multivariate analyses of factors affecting DBC are shown in Table 5. Multivariate analysis revealed that independent factors affecting DBC were CS <5 (odds ratio: 4.31, 95% confidence interval 2.39–7.78, *p* < 0.01) and trainees’ ERCP experience <3 years (odds ratio: 2.15, 95% confidence interval: 1.17–3.95, *p* = 0.01; Table 5).

**TABLE 5:** Univariate and multivariate analyses of factors affecting difficult biliary cannulation.

	Univariate analysis		Multivariate analysis	
	**OR (95% CI)**	***p*‐value **	**OR (95% CI)**	***p*‐value**
Disease				
Malignant	1.52 (0.85–2.73)	0.16	1.42 (0.75–2.67)	0.28
Benign	1		1	
Macroscopic appearance of the MDP				
Type 2 or 3	1.85 (0.90–3.83)	0.10	0.98 (0.44–2.19)	0.95
Type 1 or 4	1		1	
Peripapillary duodenal diverticulum				
Yes	1.26 (0.72–2.19)	0.42		
No	1			
Trainees’ ERCP experiences				
<3 years	2.41 (1.38–4.19)	<0.01^*^	2.15 (1.17–3.95)	0.01^*^
≥3 years	1		1	
Initial method of BC				
Contrast‐assisted	0.85 (0.42–1.70)	0.64		
Wire‐guided	1			
Comprehension score				
<5	4.60 (2.61–8.11)	<0.01^*^	4.31 (2.39–7.78)	<0.01^*^
≥5	1		1	
E‐learning				
Before	1.29 (0.76–2.19)	0.35		
After	1			

Abbreviations: BC, biliary cannulation; CI, confidence interval; ERCP, endoscopic retrograde cholangiopancreatography; MDP, major duodenal papilla; OR, odds ratio; *p < 0.05.

### Correlation of difficult biliary cannulation with comprehension scores

**FIGURE 4:**
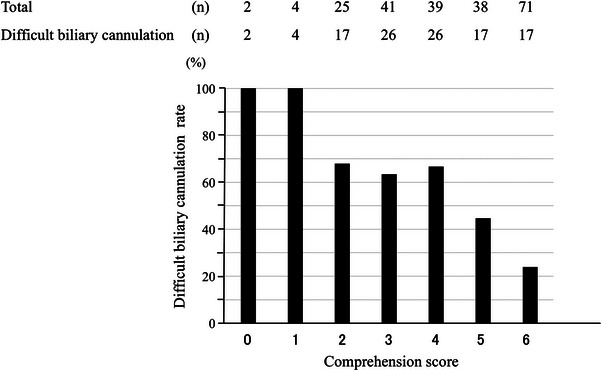
Results of the Cochran–Armitage test for trends. Negative correlation between the comprehension score and the rate of difficult biliary cannulation (*p *< 0.01).

The DBC incidence was 100% (2/2) at CS 0, 100% (4/4) at CS 1, 68% (17/25) at CS 2, 63% (26/41) at CS 3, 66% (26/39) at CS 4, 44% (17/38) at CS 5, and 24% (17/71) at CS 6. The DBC incidence showed a negative correlation with CS (*p *< 0.01; Figure 4).

### Post‐training questionnaire survey of trainees

Overall satisfaction with the program, rated on a scale of 1–10, was high, with a median score of 8 (range: 7–10). Knowledge and skills improvement were rated on a scale of 1–5 (1: “not improved at all”; 2: “improved very little”; 3: “improved slightly”; 4: “improved moderately”; 5: “improved sufficiently”). The median scores for improvement in knowledge and skills were 5 (range: 3–5) and 4 (range: 3–5), respectively. Participants viewed the training videos a median of 2 (range: 1–4) times. Feedback from trainees indicated that the e‐learning program was perceived as valuable for improving biliary cannulation skills. However, some comments noted that the videos lacked sufficient explanations regarding specific handling techniques for the endoscope and ERCP catheter.

## DISCUSSION

Video content for e‐learning focused on biliary cannulation was created and its educational effect was prospectively evaluated with 11 ERCP trainees. The DBC rate, TF rate, and AE incidence did not significantly decrease after e‐learning. However, e‐learning contributed to an improvement in understanding biliary cannulation. In addition, CS < 5 was an independent risk factor for DBC, and CS was negatively correlated with DBC rate.

Traditionally, textbooks, classroom lectures, and hands‐on seminars using simulators have been used to acquire endoscopic knowledge and techniques. Textbooks remained an effective tool for acquiring endoscopic knowledge even during the COVID‐19 pandemic. Recently, textbooks with additional video content have been marketed, thereby increasing their educational value. In contrast, face‐to‐face hands‐on training, which was limited by the recent COVID‐19 pandemic, may be effective for improving endoscopic techniques. We produced case‐based educational videos for e‐learning programs for ERCP trainees; this was aimed at enabling trainees to study anywhere, in any situation, without the need for human contact. E‐learning has shown promise as a training tool in endoscopy education. Yao et al. conducted an international randomized controlled trial and showed that an e‐learning system is effective for increasing the endoscopic detection of gastric cancer with white light images.^17^ In addition, Nakanishi et al. conducted a multicenter randomized controlled trial in Japan and demonstrated an e‐learning system to improve practitioners’ capabilities in diagnosing early gastric cancer using magnifying narrow‐band imaging.^18^ These studies have shown that e‐learning improves trainees’ understanding of endoscopic findings; however, it remains obscure that e‐learning can improve endoscopic techniques. Therefore, we undertook a novel study with the improvement of endoscopic techniques by e‐learning as the primary outcome. To our knowledge, this is the first multicenter prospective study to evaluate the educational effect of e‐learning on biliary cannulation technique improvement.

Biliary cannulation involves two processes. The first step involves identifying the bile duct opening and predicting the course of the bile duct. The second step involves manipulating the endoscope and ERCP catheter for biliary cannulation. E‐learning with video content allows trainees to efficiently simulate various types of cases, which may help them organize their knowledge and improve the first steps of biliary cannulation. However, the second step involves endoscopic techniques and may be difficult to improve through e‐learning alone. In the present study, e‐learning did not reduce DBC and TF rates, indicators of biliary cannulation technique. However, e‐learning increased comprehension scores. Additionally, CS <5 was an independent risk factor for DBC, and CS was negatively correlated with DBC rate. This result suggests that knowledge improvement is an important factor in enhancing the biliary cannulation technique. Recognizing the bile duct opening and understanding the frontal view of the MDP is presumed to increase the accuracy of approaches to the MDP and contribute to technical improvements, such as a reduction in DBC rate, in the long term. However, within the scope of this study, knowledge acquisition alone did not lead to considerable improvement in the biliary cannulation technique. In other words, practical training in the manipulation of the endoscope and ERCP catheter may be essential for efficient biliary cannulation training. Previous studies have demonstrated that mechanical simulators are useful for learning biliary cannulation.^6^, ^7^, ^8^, ^9^ Combining e‐learning with mechanical simulators has the potential to enhance learning efficiency, and further research is warranted to advance biliary cannulation training.

In the present study, e‐learning did not contribute to the reduction in AE incidence either. A prospective multicenter study showed that trainee involvement was a risk factor for post‐ERCP pancreatitis (PEP).^19^ Trainees should achieve technical improvement while reducing AEs; this is an important issue in endoscopic education.

This study had several limitations. First, this is not a comparative study with or without an e‐learning program; thus, there may have been unintentional bias. However, since we believe that all trainees deserve equal access to education, e‐learning was provided only to trainees who wished to learn in actual clinical practice. Second, this study included a relatively small sample size of 10 cases to assess comprehension and technical outcomes before and after e‐learning. To minimize the influence of skill improvement due to the accumulation of procedural experience, the pre‐ and post‐learning assessments were intentionally limited to 10 cases. Third, the primary outcome as a technical indicator of the biliary cannulation technique was defined on the basis of the 5‐2‐2 criterion.^13^ However, DBC has multiple definitions.^20^, ^21^ The 5‐5‐2 criteria were adopted in the present study because they are associated with PEP incidence.^13^ Adopting different criteria may result in different outcomes. Fourth, this study did not restrict trainees from performing biliary cannulation for patients with non‐native papillae during the study period. This may have influenced the accuracy of the e‐learning evaluation. Finally, according to the feedback from the trainees, our video lacked detailed instructions on how to handle endoscopes and ERCP catheters. Adding content on such topics to the video may improve its educational effectiveness.

In conclusion, e‐learning with video content did not contribute to the improvement of trainees’ biliary cannulation technique. However, e‐learning improved the understanding of biliary cannulation, suggesting its potential to support the future acquisition of biliary cannulation skills.

## CONFLICT OF INTEREST STATEMENT

None.

## ETHICS STATEMENT

**Approval of the research protocol by an Institutional Reviewer Board**: The study was approved by the institutional review board of each participating centre.

## PATIENT CONSENT STATEMENT

Written informed consent was obtained from all endoscopy trainees who participated in this study. Written informed consent was also obtained from the patients for ERCP, and consent for the use of patient information was obtained through an opt‐out system.

## CLINICAL TRIAL REGISTRATION

The study was registered in the University Hospital Medical Information Network Trials Registry (UMIN000044223).

## Supporting information



SUPPLEMENTAL VIDEO 1.mp4

## Data Availability

The data that support the findings of this study are available on request from the corresponding author, Junichi Kaneko. The data is not publicly available due to restrictions, for example, their containing information that could compromise the privacy of research participants.
